# A Hybrid Deep Autoencoders and Random Forest Framework for False Data Injection Attack Detection in Industrial Internet of Things Networks

**DOI:** 10.3390/s26165110

**Published:** 2026-08-12

**Authors:** Abdullah M. Albarrak, Fuad A. Ghaleb, Sultan Noman Qasem, Faisal Saeed

**Affiliations:** 1 Computer Science Department, College of Computer and Information Sciences, Imam Mohammad Ibn Saud Islamic University (IMSIU), Riyadh 11432, Saudi Arabia; amsbarrak@imamu.edu.sa (A.M.A.); snmohammed@imamu.edu.sa (S.N.Q.); 2 Department of Computer Science, Birmingham City University, Birmingham B4 7XG, UK; faisal.saeed@bcu.ac.uk

**Keywords:** false data injection, FDIA, hybrid model, Industrial Internet of Things, IIoT, adversarial attacks, stealthy attack detection

## Abstract

The rapid adoption of Internet of Things (IoT)-enabled applications has significantly expanded the cyberattack surface across a wide range of critical systems such as industrial IoT (IIoT), smart grids, transportation, healthcare, industrial control systems, and smart cities. False data injection attack (FDIA) has emerged as a serious security threat to these applications due to its stealthiness and adversarial nature, silently corrupting the data integrity of critical operational processes without triggering conventional detection mechanisms. Existing FDIA solutions rely on single-model architectures that are built based on classical or limited predefined attack scenarios. Such solutions often fail to achieve robust detection under adversarial and evolving attack conditions; accordingly, they lack generalisability and are insufficient to capture the broader scope of FDIAs. In this study, a hybrid detection framework is proposed that integrates a Random Forest classifier with an unsupervised anomaly detection model based on a deep autoencoder combined through a Logistic Regression metaclassifier. The proposed framework addresses the gap in single-model detectors that either rely on fixed decision boundaries that struggle with gradually evolving stealthy FDIA patterns or on anomaly detection that lacks strong discriminative power in separating subtle adversarial deviations from normal operational variability. Different types of stealthy and adversarial FDIA have been modelled and injected into the dataset samples for use in training the proposed model. The results show that the overall detection performance of the proposed architecture improved by 2.39 percentage points in terms of F1-score while maintaining a low false-positive rate of 0.49%. These findings reflect the effectiveness of feature representation learning via autoencoders and hybrid classification strategies against stealthy and adversarial FDIA patterns. Future work should include temporal modelling for further advancing robust detection against evolving adversarial threats.

## 1. Introduction

Internet of Things technology development has grown rapidly over the last decade and is expected to experience significant growth over the next decade. According to Market Research Future [1], the global market of IoT is expected to grow from 193 billion USD in 2025 to 1.43 trillion in 2035. A study in IoT Analytics [2] stated that the number of connected IoT devices is expected to grow from 21.1 billion in 2025 to 50 billion by 2035. The increasing adoption of IoT devices has been driven by the rapid development of Artificial Intelligence, the automation demands, and the advancement of communication technologies. Many smart programmes have been studied, including smart cities, smart homes, power plants, smart industry, smart grids, transportation systems, and health care, aiming at improving efficiency and resource utilisation, reducing operational costs, improving productivity, and enabling real-time monitoring and accurate decision-making. These technologies rely on the trustworthiness of the information collected by sensors, as they will be used for critical decision-making impacting people’s lives and properties. However, IoT applications are subjected to numerous kinds of security threats that hinder their deployment and prevent these applications from achieving their intended goals.

False Data Injection Attacks (FDIAs) are a significant concern for cyber-physical systems (CPS), including IoT, emerging IIoT networks, and Operational Technology (OT) environments, as they can compromise data integrity, interfere with operations, and lead to incorrect critical decisions that may cause severe physical and economic damage, including harm to people’s lives and properties [3,4,5]. Several high-profile FDIA incidents have demonstrated the impact of data manipulation attacks on industrial systems. For instance, in 2009, the Stuxnet worm targeted Siemens industrial control systems (ICS) in the United States before later disrupting parts of Iran’s nuclear development infrastructure in 2010 [6]. Recently, in December 2015, approximately 225,000 customers in Ukraine suffered a power outage lasting several hours due to an FDIA targeting substation circuit breakers [7,8]. These incidents highlight the critical importance of maintaining data integrity in interconnected industrial environments. With the increasing adoption of IIoT technologies, industrial systems are becoming more interconnected and exposed to similar threats. In IIoT networks, attackers may either manipulate sensors to generate false data or inject malicious data through compromised hosts. In both cases, the attacker generates falsified measurements that are carefully crafted, usually within the normal space of the network, to remain orchestrated to evade detection and mislead detection models [7,9].

FDIA detection mechanisms have been extensively reviewed in the literature [7,9,10,11,12] and can be categorised into three main approaches: physical or model-based [5,13,14,15,16], statistical-based [13,17,18], and machine learning-based methods [6,19,20,21,22,23]. Physical-based approaches focus on validating the consistency of measurements against expected values generated through system state estimation models, redundant sensors, or digital twins. These approaches are effective in detecting simple FDIA; however, coordinated and gradual shift FDIAs within a plausible range can remain stealthy, rendering such models ineffective for detection. In addition, these solutions require domain expertise and accurate system models, which are challenging to obtain due to the time-varying, nonlinear, and dynamic characteristics of real-world systems, thereby reducing reliability and increasing false alarms. Statistical-based methods such as in [13,17,18] assume static data distributions and expected statistical behaviour, relying on predefined thresholds for anomaly detection. Although statistical techniques can effectively thwart classical blind and random FDIAs, stealthy and adversarial FDIAs can exploit these limitations and remain undetected. Machine learning techniques learn patterns from historical data and can effectively detect FDIA. Many studies, such as in [4,6,19,20,21,22,23], show that deep learning techniques such as Deep Neural Networks (DNNs) and Convolutional Neural Networks (CNNs) show strong discriminative performance against FDIA. However, machine learning models, especially neural networks, are themselves vulnerable to adversarial attacks. Attackers can generate false data that camouflages itself as normal data or orchestrate attacks designed to minimise the anomaly score, thereby evading detection.

Several data-driven approaches use different kinds of machine learning techniques, aiming for effective FDIA detection. The current literature has demonstrated that deep learning models, particularly DNNs, CNNs, LSTM networks, and autoencoders, offer strong capabilities for FDIA detection due to their effectiveness in learning complex nonlinear relationships and temporal dependencies inherent in IIoT measurement data [3,4,6,19,21,23,24,25]. However, most existing solutions are trained using sparse FDIAs samples and primarily focus on conventional attack patterns, which limits their generalisation capability against stealthy and adversarial FDIA variants [26]. Few studies show the effectiveness of autoencoders for identifying anomalous measurement behaviour associated with stealthy FDIA through unsupervised reconstruction-based learning [6,27]. However, existing autoencoder-based solutions lack strong discriminative decision boundaries, leading to a high false alarm rate and degrading the reliability of the solutions. Similarly, tree-based algorithms such as Decision Tree, Random Forest, Extra Trees, and XGBoost have demonstrated competitive FDIA detection performance owing to their robustness in handling nonlinear, high-dimensional, and heterogeneous IIoT data while maintaining strong generalisation capability and resistance to overfitting [6,13,22,28]. However, these solutions rely on a single model that stems from limited and sparsely known attack patterns. In addition, tree-based solutions rely on the original features and do not learn latent representations, leading to reduced sensitivity to subtle distributional shifts, resulting in poor detection of stealth and gradual FDIA. In addition, existing research treats intrusion detection and FDIA detection as separate tasks, which is not realistic in a challenging cyber threat environment.

To address these limitations, this study proposes a hybrid ensemble framework based on deep autoencoder (AE), Random Forest (RF), and Logistic Regression (LR) techniques for detecting false data injection attacks in IIoT environments. The Random Forest component learns known FDIA patterns by constructing non-linear discriminative decision boundaries that enhance its ability to discriminate between normal and falsified measurements while remaining robust against adversarially crafted FDIA attempts. Unlike conventional single-model approaches, the proposed framework incorporates a deep autoencoder as an unsupervised anomaly detector to complement the Random Forest classifier. The autoencoder learns compact latent representations. Logistic Regression (LR) is employed for decision-making through learning from the class probability output of the RF and the latent and reconstructed representations generated by the AE, thereby maximising overall detection performance while minimising false alarms. To improve generalisability and better represent real-world threat environments, both network-level intrusions and FDIAs were incorporated into the training of the Random Forest classifier, unlike most existing studies that consider these threats separately. Several types of stealthy and adversarial attack models were modelled, simulated, and injected into the TON_IoT dataset to resample realistic FDIAs.

The rest of this paper is organised as follows: Section 2 reviews the related work, Section 3 presents the FDIA model, and Section 4 describes the methods and methodology of the proposed solution. Section 5 outlines the dataset and evaluation procedures, Section 6 presents the results and discussion, and Section 7 provides the conclusion and future work.

## 2. Related Work

False data injection attacks have been extensively studied across a broad range of IoT application domains, including smart grids, industrial control systems, cyber-physical systems, and smart cities. Several dedicated surveys consistently identify FDIA as one of the most critical and technically challenging data integrity threats facing modern connected infrastructure [7,9,10,11,12]. Authors in [12] presented a review of FDIA models, targets, and their impact on cyberspace. A comprehensive survey in [10] examined machine learning-based approaches for detecting false data injection attacks in smart grid systems. Nand et al. [11] highlighted that data-driven approaches, especially machine learning techniques, are widely considered effective for FDIA detection because they can identify complex patterns in data without depending on predefined rules or precise physical models. Their survey reviewed several ML methods, including Support Vector Machines (SVM), Random Forests (RF), K-Nearest Neighbors (KNN), Artificial Neural Networks (ANN), Deep Neural Networks (DNN), autoencoders (AE), Convolutional Neural Networks (CNN), Recurrent Neural Networks (RNN), and hybrid deep learning models. Irfan et al. [29] present a systematic survey of the detection and localisation of false data injection attacks in smart grids, highlighting how FDIA can bypass conventional Bad Data Detection by compromising the state estimation process and identifying joint detection and localisation as a critical open challenge. However, the survey focuses exclusively on the smart grid domain and does not address FDIA in broader IoT and IIoT environments. Alsabbagh et al. [30] demonstrate how attackers can exploit communication and control-system vulnerabilities in IIoT environments to inject malicious data and conceal their activities. However, the study focuses primarily on attack construction and exploitation rather than detection and does not consider stealthy FDIA variants designed to remain within the statistical plausibility range of normal sensor behaviour.

Shees et al. [22] demonstrated that machine learning can significantly improve the early detection of FDIAs. Their study evaluated several machine learning techniques, including Extra Trees Classifier (ETC), RF, XGBoost, Logistic Regression (LR), Decision Tree (DT), and Bagging Classifier (BC). The results showed that ensemble tree-based methods achieved the best performance among the evaluated models. Xu et al. [23] evaluated several machine learning algorithms for FDIA detection, including Logistic Regression, SVM, DNNs, CNNs, and Recurrent Neural Networks (RNNs). These supervised models were trained to classify measurements as either normal or compromised. The authors further proposed a DNN-based framework with random input padding to enhance robustness against adversarial measurements.

Nagaraj et al. [13] proposed an Ensemble Correlation-based Detector with Adaptive Statistics (ECD-AS) for FDIA detection. The framework uses sliding-window adaptive mean, covariance, and anomaly thresholds to capture spatial and temporal variations in system behaviour. However, the method relies heavily on correlation structures that may not remain valid under coordinated or stealthy attacks designed to preserve state estimation consistency. A study in [31] proposed an Extreme Learning Machine (ELM)-based One-Class-One-Network (OCON) framework for FDIA detection in smart grids.

Potluri et al. [32] used an ANN to develop a detection model for identifying FDIAs injected into data generated from a simulated industrial control system. He et al. [4] combined a State Vector Estimator (SVE) with a Deep Belief Network (DBN) to extract high-dimensional temporal features for real-time FDIA detection. Wei et al. [25] employed a Deep Belief Network to detect FDIAs and reported higher detection accuracy compared with SVMs. An SVM-based residual detection framework was proposed by [33] for false data injection attacks in industrial control systems, using prediction models to detect deviations between measured and estimated sensor values and replace compromised data. However, the approach mainly evaluates scaling-based FDI attacks and does not explicitly consider stealthy or adaptive adversarial attack strategies, such as carefully crafted injections designed to evade residual-based detection. Saurabh et al. [34] propose a hybrid machine-learning framework combining MLP for automatic feature extraction and XGBoost for classification to detect FDI attacks in IIoT environments using the Edge-IIoTset dataset across multiple attack scenarios and protocols. However, the work focuses primarily on general network intrusion detection rather than stealthy and adversarial FDIA detection.

Hao et al. [35] proposed an unsupervised detection approach based on Robust Principal Component Analysis (RPCA). Their method assumes that normal measurement data exhibits a low-rank structure, while malicious measurements are sparse. However, the approach may be less effective against coordinated or dense FDIAs in which attackers manipulate a large number of measurements simultaneously, thereby reducing attack sparsity. Nagaraj et al. [13] proposed a deep autoencoder-based anomaly detection framework to learn latent representations of normal system behaviour for FDIA detection in smart grids. Although the method achieved promising detection performance, it requires extensive historical training data and may remain vulnerable to adversarial FDIAs that closely resemble legitimate measurements. Aboelwafa et al. [36] proposed an autoencoder-based approach for detecting FDI attacks in a hydraulic sensor-based system, exploiting hidden correlations in sensor measurements to identify attacks and using a denoising autoencoder to recover corrupted data. The approach outperformed SVM-based methods across two FDI attack scenarios; however, the study does not distinguish between basic and stealthy FDIA variants. Hafizunisa et al. [20] introduced an autoencoder-based approach to identify inconsistencies between corrupted data patterns and the expected correlation structures learned from normal operational data. Majidi et al. [6] combined a stacked autoencoder for dimensionality reduction with an Extremely Randomised Trees (Extra Trees) classifier to improve FDIA detection accuracy while addressing data sparsity and feature complexity. Nevertheless, the attack representation mainly focused on structured residual-preserving attacks, limiting robustness against adaptive, coordinated, low-sparsity, or adversarial FDIAs that closely mimic legitimate measurement distributions. Deep learning models, particularly DNNs, CNNs, LSTMs, and autoencoders, were reported as effective for detecting many kinds of FDIA due to their capabilities in learning complex nonlinear and temporal patterns [11,19]. Deep autoencoders (AEs) were frequently reported as an effective unsupervised approach to defend against FDIA [13,22]. Tree-based algorithms have been reported as effective for detecting FDIAs due to their capability of handling the nonlinear and high-dimensional characteristics [11,22]. The study in [37] proposes an FDIA detection framework for IIoT using a statistical and information-theoretic selection (SITS) technique for feature selection and a hybrid deep learning ensemble of TCN, DBN, and AE to identify malicious sensor data manipulation. However, it focuses on predefined FDIA patterns and does not address stealthy attack generation or adversarial strategies for evading detection. Ashrafuzzaman et al. [19] compared a DNN with Gradient Boosting Machines (GBM), Generalized Linear Models (GLM), and Distributed Random Forests (DRF) for FDIA detection. However, the attack model focused only on static state estimation and did not consider dynamic grid behaviour or coordinated attack scenarios.

Authors in [38] introduce the UKMNCT_IIoT_FDIA dataset, which contains a range of false data injection attack scenarios for classifying, identifying, and detecting false-data-injection attacks in Industry 5.0 IIoT settings. However, the dataset mainly supports the development and evaluation of FDIA detection algorithms using ML/DL-based learning and does not specifically investigate stealthy or adversarial attacks designed to evade AI-based detection models. The study in [39] proposes a consensus-based intrusion detection and mitigation mechanism that combines watchdog surveillance with collaborative consensus to exclude FDI attacks. However, the approach mainly targets data validation and network-level attack mitigation and does not explicitly address sophisticated stealthy FDIA where manipulated data remain close to normal system behaviour. Orman [40] proposes an IDS framework integrating machine learning, deep learning, and hybrid models for cyberattack detection in IIoT SCADA environments, evaluating architectures including decision trees, random forests, CNNs, and MLP on the WUSTL-IIoT-2021 dataset. However, the work focuses on DoS and DDoS attacks and does not address false data injection.

Authors in [41] present an approach for detecting false data injection and man-in-the-middle attacks in IoT and IIoT environments using machine learning-based detection mechanisms. However, the evaluation mainly focuses on attack detection performance and does not deeply investigate adversarial attack generation or stealthy FDIA scenarios designed to evade detection. The study in [21] proposes a privacy-preserving FDIA detection framework for smart grids by integrating Transformer-based detection, federated learning, and Paillier cryptography. However, it focuses on detection performance and secure data sharing, without explicitly addressing stealthy attack generation or adversarial evasion strategies. Authors in [42] propose a false data injection attack that exploits MQTT vulnerabilities to manipulate IIoT camera feeds and deceive system operators. However, the attack is limited to false visual data injection through compromised camera streams and does not consider attacks on other sensor modalities. Liu et al. [36] propose a tensor-completion-based FDI attack algorithm for IoT-enabled smart grids that manipulates compromised measurements to match historical patterns, achieving stealthiness against both statistical and ML-based detectors. However, the approach is specifically designed for smart-grid state estimation and does not address stealthy FDIA in broader IIoT environments with heterogeneous sensor data. Ran and Ma [43] propose an extended FDIA model for cyber-physical power systems that integrates additional load attack components into state variables through an optimisation-based approach, maintaining stealthiness against both Bad Data Detectors and neural attack detection schemes. They also develop a Deep Q-Network-based detection framework to reduce detection delays and false alarms. However, the work remains focused on smart-grid state estimation and does not consider FDIA detection across broader IoT and IIoT environments or heterogeneous attack scenarios.

Many studies focus primarily on static state estimation models, whereas practical smart grid environments operate under dynamic, time-varying, and large-scale conditions that are considerably more complex and vulnerable to sophisticated cyberattacks [27,44]. Moreover, most existing machine learning and deep learning models are designed to detect basic FDIAs in which malicious measurements exhibit clear statistical deviations from normal operating conditions. Consequently, their robustness against stealthy, adaptive, and adversarial FDIAs, where attackers deliberately craft malicious data to closely resemble legitimate measurements and evade detection, remains insufficiently investigated. Recent studies have shown that deep learning techniques, particularly DNNs, CNNs, LSTM networks, and AEs, are effective for FDIA detection due to their capabilities to effectively learn nonlinear relationships and temporal dependencies inherent in smart grid measurements [6,11,19]. Deep autoencoders have received considerable attention as an effective unsupervised mechanism for identifying anomalous measurement behaviour associated with stealthy FDIAs by learning compact latent representations of normal operating conditions [13,22]. However, relying solely on unsupervised reconstruction-based approaches may result in limited discrimination between sophisticated attacks and legitimate operational variations, particularly when malicious measurements closely resemble normal data distributions. In addition, these models are generally evaluated using conventional FDIA datasets and may exhibit reduced effectiveness when confronted with adaptive or adversarial attacks that closely mimic legitimate operational patterns. Similarly, ensemble tree-based algorithms such as Random Forest, Extra Trees, and XGBoost have also demonstrated strong FDIA detection performance due to their robustness in handling nonlinear, high-dimensional, and heterogeneous smart grid data while maintaining strong generalisation capability and resistance to overfitting [11,22]. Nevertheless, supervised tree-based approaches typically depend on labelled attack data and may struggle to detect previously unseen or stealthy attack behaviours. Recent studies indicate that existing FDIA research remains fragmented across attack models, detection methods, and application domains. While some studies address stealthy FDIA in smart-grid state estimation [29,36,43], others focus on specific FDI scenarios or general IIoT intrusion detection [34,45]. However, limited attention has been devoted to systematically evaluating detection robustness across progressively sophisticated FDIA scenarios within heterogeneous IIoT environments. In particular, the effect of increasing attacker capability, from conventional FDIA to statistically constrained stealthy and detector-aware adversarial attacks, on the detection of common FDIA morphologies remains insufficiently investigated. This limitation is particularly relevant because the statistical plausibility of injected measurements can directly affect the ability of ML-based detectors to distinguish malicious manipulations from legitimate operational variations.

Therefore, this study designs and develops a complementary hybrid detection framework that integrates the discriminative classification capability of a tree-based Random Forest classifier with the unsupervised anomaly detection capability of a deep autoencoder. Unlike most existing studies, this work explicitly models conventional, stealthy, and adversarial FDIA scenarios to simulate a wider range of realistic attack behaviours. The proposed framework is then comprehensively evaluated against these attack models to assess its detection accuracy and robustness under sophisticated attack conditions. This approach addresses the limitations of single-model methods and provides more robust and comprehensive detection coverage against stealthy and adversarial FDIA vectors.

## 3. FDI Attack Model

Numerous studies have demonstrated how False Data Injection can bypass traditional detection mechanisms, including the conventional Bad Data Detection (BDD) schemes and machine learning-based Intrusion Detection Models [22]. These attacks exploit the mathematical properties of the state estimation process to manipulate measurement data while preserving the statistical characteristics used by conventional detectors. It has been shown that an adversary can evade detection by creating stealthy attacks if the adversary possesses knowledge of the system topology and/or design. A comprehensive survey of FDIA techniques in smart grids has been reviewed by Liang et al. [46]. In their survey, the authors demonstrated how stealthy and valid FDIA can be constructed under different levels of attacker knowledge regarding network topology, detection mechanisms, and system configuration. In this study, to demonstrate how stealthy and adversarial FIDA manipulates measurement data within IIoT environments to inject consistent but false data into the system model.

Let z denote the original measurement vector, x the system state, h(x) the nonlinear measurement function, and e the uncertainties or the measurement noise. The relationship between the system state x and the measured observations z can be expressed as

(1) $$z\approx h\left(x\right)+e\;$$

Equation (1) represents the nonlinear measurement model used in state estimation, where the measurements are generated as a function of the system state and are affected by measurement noise. For practical state estimation, the nonlinear measurement function h(x) is linearized at the specific point $x_{0}$ using a first-order Taylor series expansion, yielding:

(2) $$h\left(x\right)\approx h\left(x_{0}\right)+H\left(x-x_{0}\right)$$

where H is the Jacobian matrix that describes the sensitivity of the measurements to changes in the system state.

(3) $$H=\frac{\partial h(x)}{\partial x}\;$$

Then

(4) z=Hx+e 

To simulate the classical false data $z_{cla}$, the attacker injects false data vector as follows:

(5) $$z_{cla}=z+\delta \;$$

where δ denotes the injected false data perturbation. To simulate stealthy attack $\delta_{s}$ assuming attacker know the H then

(6) $$a_{stl}=Hc\;$$

where c is the spoofed state perturbation vector which is crafted to mislead the state estimation to generate false but stealthy measurements. By constructing the attack vector in the column space of the Jacobian matrix, the injected measurements remain consistent with the state estimation model, thereby preserving the residual used by BDD schemes. Then, the stealthy attack $z_{stl}$ can be represented as follows:

(7) $$z_{stl}=H\left(x+c\right)+e\;$$

where the statistics of the residual $r_{stl}\;$ remain unchanged as follows.

(8) $$r_{stl}=z_{stl}-H\left(x+c\right)=e\;$$

Since the attack does not alter the residual distribution, conventional residual-based Bad Data Detection methods cannot distinguish the attacked measurements from normal measurement noise. In coordinated stealthy attacks, multiple sensors or measurement groups are compromised simultaneously to launch a synchronised attack against the state estimation process. The attack spans several vectors. The overall attack vector is constructed as the sum of multiple stealthy attack vectors and is expressed as follows:

(9) $$\delta_{cor}=\sum_{i=0}^{m}H_{i}c_{i}$$

where m denotes the number of compromised measurement groups or attack sources, $H_{i}$ is the Jacobian matrix associated with the $i^{\mathrm{th}}$ measurement group, and $c_{i}$ is the corresponding state perturbation vector introduced by the attacker. The resulting attack vector preserves the physical consistency of the measurements while simultaneously affecting multiple locations in the system, making the attack more difficult to detect using conventional residual-based detection methods. Dynamic stealthy attacks $a_{k}$ evolve over time by adapting the attack vector at each sampling instant, enabling the attacker to evade detectors that exploit temporal correlations, such as Kalman filter-based state estimators.

(10) $$\delta_{k}=Hc_{k}$$

where $c_{k}$ evolve each time epoch to exploit state estimation-based solutions such as Kalman filters. Adversarial FDIA extends classical FDIA by adding carefully crafted perturbation to slightly modify the measurements to fool the machine learning classifiers as follows.

(11) $${min}_{\delta }\;\left\|\delta \right\|\;subject\;to\;f(z+\delta )=Normal$$

where δ denotes the adversarial perturbation with minimum norm, and f(⋅) represents the target machine learning classifier. The optimisation seeks the smallest perturbation that causes the classifier to incorrectly classify an attacked measurement as normal.

## 4. The Proposed Solution

**Figure 1:**
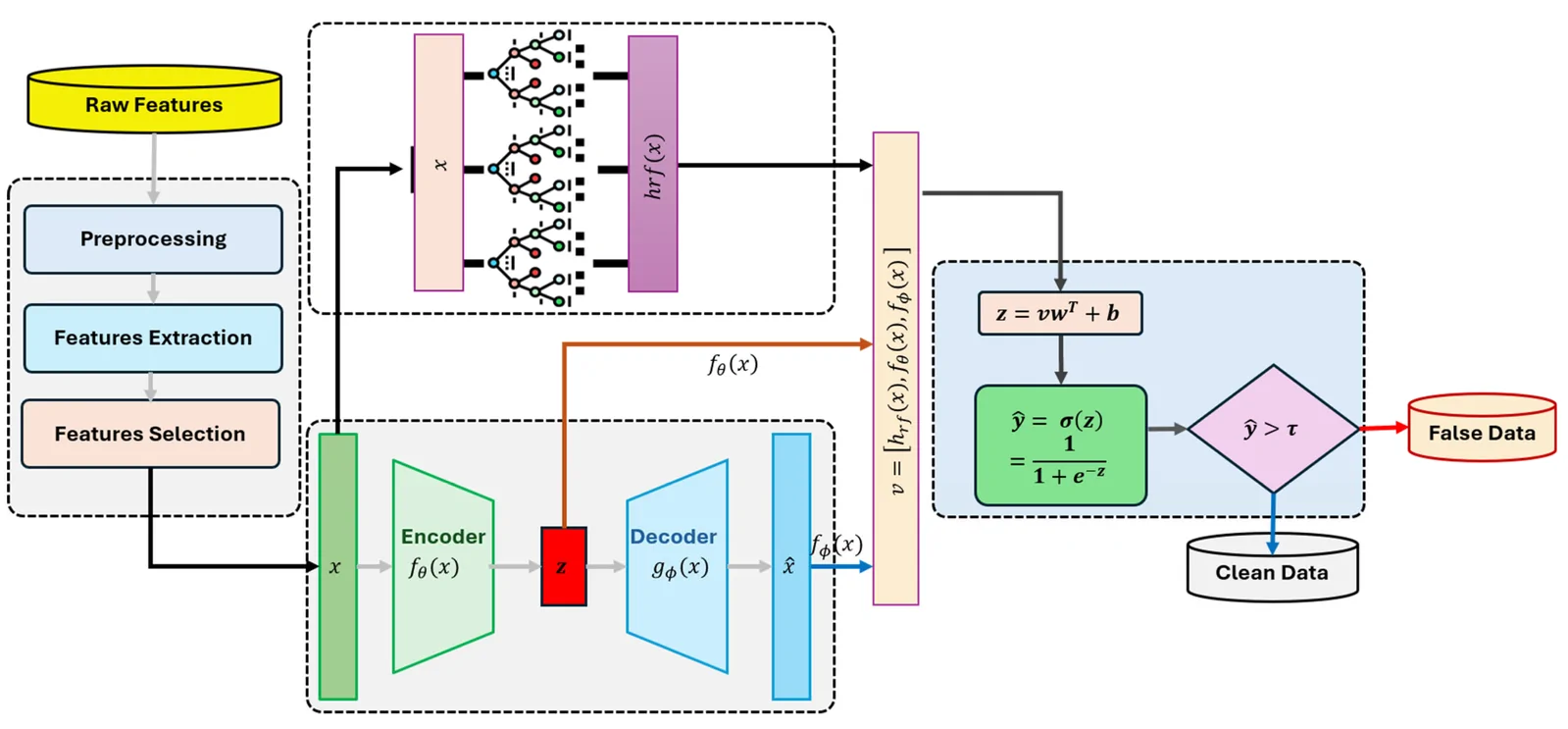
The proposed hybrid autoencoder and random forest framework for FDIA detection.

This section presents the proposed hybrid model for detecting stealthy and adversarial FDIAs. As shown in Figure 1, both supervised and unsupervised deep learning approaches were integrated into the proposed framework by combining a Random Forest (RF) classifier, a deep autoencoder (AE), and a Logistic Regression (LR) meta-classifier. The RF algorithm is constructed as an ensemble of decision tree classifiers, each trained with a bootstrap subset of features [22,24,28]. RF is an effective discriminative classifier that has been employed in many solutions for detecting cyber-attacks. The underlying intuition is that Random Forest induces a discrete, piecewise-constant partitioning of the feature space, requiring FDIA samples to traverse multiple non-smooth decision boundaries to evade detection. Furthermore, because Random Forest does not explicitly capture temporal dependencies and treats samples as independent, time-evolving attack patterns may become detectable once they induce sufficient distributional drift across successive observations. Let D as the dataset contains N number of historical samples $x_{i}$ of normal and attack with label $y_{i}$

(12) $$D={\left\{\left(x_{i},y_{i}\right)\right\}}_{i=1}^{N}\;$$

The RF algorithm consists of an ensemble of decision trees, each trained using a bootstrap sample of the training data D and a randomly selected subset of features. It creates B bootstrap sets of datasets by random sampling features with replacement.

(13) $$D_{b}\;\subset \;D\;:\;b=1,2,\ldots ,B\;$$

Each bootstrap set $D_{b}$ is used to train one decision tree. For each feature $f_{j}$ in the $D_{b}$ the RF algorithm search for feature $f_{j}$ and threshold s that minimise the impurity G(t) at node t.

(14) $$G\left(t\right)=1-\sum_{k=1}^{K}p_{k}^{2}\;$$

where $p_{k}$ denotes the proportion of samples belonging to class k. The RF grows multiple trees. Each tree selects the best split at each node by finding the split s that minimise the weighted impurity $G_{s}$ as follows:

(15) $${argmin\;G}_{s}(s,\;j)={arg\;minG}_{s}\left\{\frac{N_{l}}{N}G_{l}+\;\frac{N_{r}}{N}G_{r}\right\}\;$$

where $N_{l}$ and $N_{r}$ denote the number of samples in the left and right child nodes after the split, respectively, N is the total number of samples, and $G_{l}$ and $G_{r}$ represent the impurity of the left and right child nodes, respectively. Each tree h(x) produces a probabilistic output p(y=1∣x) = h(x) for the class of the input sample.

RF-based solutions rely on ensemble classifiers for decision-making. Due to its ensemble structure, RF effectively captures nonlinear decision boundaries and has been widely adopted for cyberattack detection [22,24,28]. Its discriminative learning capability enables it to identify attack patterns from labelled data, making it effective for detecting known FDIAs. This makes it more robust to adversarial attacks, as it is needed to make perturbations to influence a majority of independently trained trees before the final decision can be altered. Consequently, small or unstructured perturbations are often insufficient to change the overall classification outcome. However, stealthy and adversarial FDIAs are intentionally designed to closely resemble normal operational data, causing them to lie near or across learned decision boundaries. Thus, this robustness is not guaranteed in all settings, particularly under coordinated or physics-aware false data injection attacks, where structured perturbations can affect multiple correlated measurements simultaneously and increase the likelihood of consistent misclassification across the ensemble. Consequently, the discriminative capability of RF alone may be insufficient for identifying attacks that exhibit statistical characteristics like legitimate measurements.

**Figure 2:**
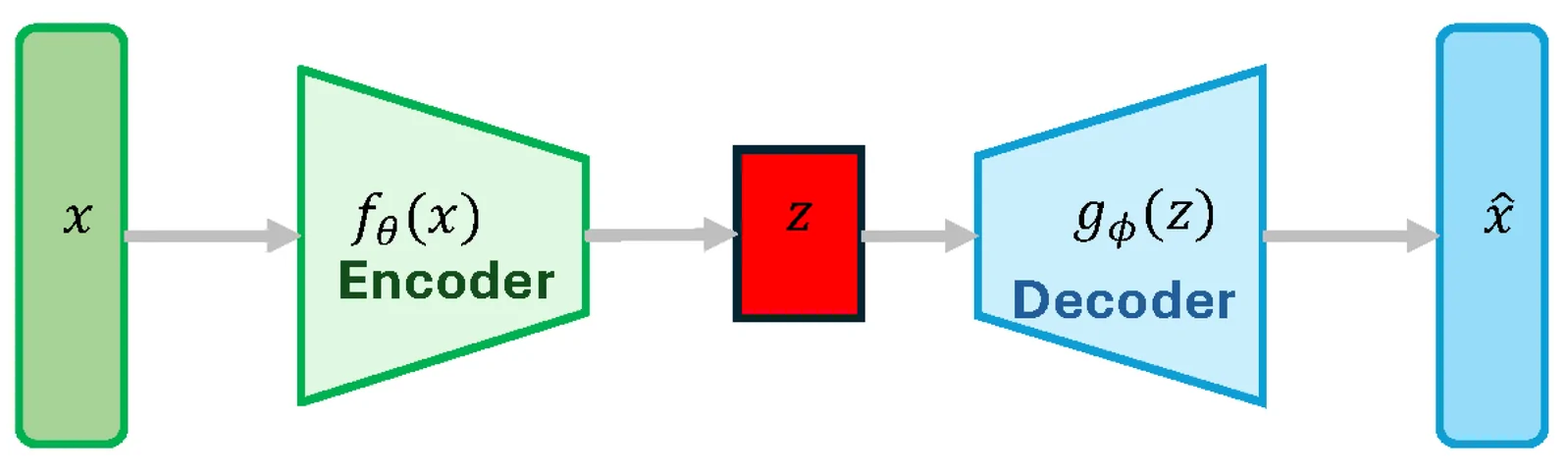
Structure of deep autoencoder.

To address this limitation, a deep autoencoder (AE) is incorporated to learn the underlying correlations, constraints, and structural patterns of normal IIoT measurements. A deep autoencoder is an unsupervised approach used to train an anomaly-based detection model by learning normal behaviours of a target system. Though it can capture the hidden correlations, nonlinear structure, and multivariate dependencies, such characteristics enable AE to construct a compact latent representation of normal operating conditions. Consequently, measurements that deviate from the learned normal distribution, such as stealthy or adversarial FDIAs, produce higher reconstruction errors and distinctive latent representations, making them more readily identifiable and widely recognised as an effective approach for anomaly detection and having demonstrated promising performance in detecting stealthy FDIAs [6,20,27,37]. Figure 2 depicts the structure of the deep autoencoder. As shown in the figure, the autoencoder consists of two layers, the encoder and decoder. The encoder layer compresses the input space features, x, into a lower-dimensional latent space z, also called the Bottleneck. This compressed representation captures the most significant patterns and underlying characteristics of the input data.

(16) $$z=f_{\theta }\;\left(x\right)=\sigma \left(W_{e}x+b_{e}\right)\;$$

where x represents the original input feature vector, z denotes the latent representation, $\sigma \left(\cdot \right)\;$ is the activation function, and $W_{e}$ and $b_{e}$ represent the weight matrix and bias vector of the encoder, respectively. The parameter set of the encoder is denoted as $\theta =\{W_{e},b_{e}\}$, which is optimised during the training process. The decoder layer tries to reconstruct the original input space features $\hat{x}$ from the learned latent representation. The reconstruction operation is defined as

(17) $$\hat{x}=g_{\phi }\;(z)=\sigma \left(W_{d}z+b_{d}\right)\;$$

where $\hat{x}$ is the reconstructed input, $W_{d}$ and $b_{d}$ are the decoder weight matrix and bias vector, respectively, and $\phi =\{W_{d},b_{d}\}$ represents the decoder parameters. The objective of the decoder is to generate a reconstruction $\hat{x}$ that is as similar as possible to the original input x.

The autoencoder is trained by minimising the reconstruction error between the original input and its reconstructed output. The commonly used loss function is the mean squared error (MSE), which is formulated as

(18) $${min}_{\theta ,\phi }L\left(x,\hat{x}\right)={min}_{\theta ,\phi }\frac{1}{N}\sum_{i=0}^{N}{\left\|x_{i}-{\hat{x}}_{i}\right\|}^{2}\;$$

where N represents the number of training samples, $x_{i}$ is the original input vector of the $i^{th}$ sample, and ${\hat{x}}_{i}$ is the corresponding reconstructed output. The optimisation process adjusts the encoder and decoder parameters θ and ϕ to minimise the reconstruction error.

Consequently, the AE learns the underlying correlations, constraints, and structural patterns of normal system operation. When a stealthy FDIA sample $x_{a}$ is introduced, it may deviate from the learned representation of normal system behaviour, resulting in an inaccurate reconstruction and an increased reconstruction loss:

(19) $$L\left(x_{a},{\hat{x}}_{a}\right)={\left\|x_{a}-{\hat{x}}_{a}\right\|}^{2}>L\left(x,\hat{x}\right)\;$$

where $x_{a}$ and ${\hat{x}}_{a}$ denote the attacked sample and its reconstruction, respectively. Therefore, the reconstruction error can be used as an anomaly score, where samples with errors exceeding a predefined threshold are identified as potential FDIA events. This occurs because the attacked sample alters the latent feature representation $z_{a}$, preventing the autoencoder from reconstructing the manipulated measurements with the same accuracy as normal operational data.

In this study, to improve the detection accuracy while mitigating stealthy and adversarial samples, the latent representations and reconstruction features extracted by the autoencoder are combined with RF probability outputs and provided to a logistic regression-based model for decision-making. This hybrid architecture enables the model to learn complex nonlinear decision boundaries directly from data. Moreover, such multi-stage design improves robustness against stealthy and coordinated FDIA by combining nonlinear representation learning, ensemble diversity, and probabilistic decision fusion.

**Figure 3:**
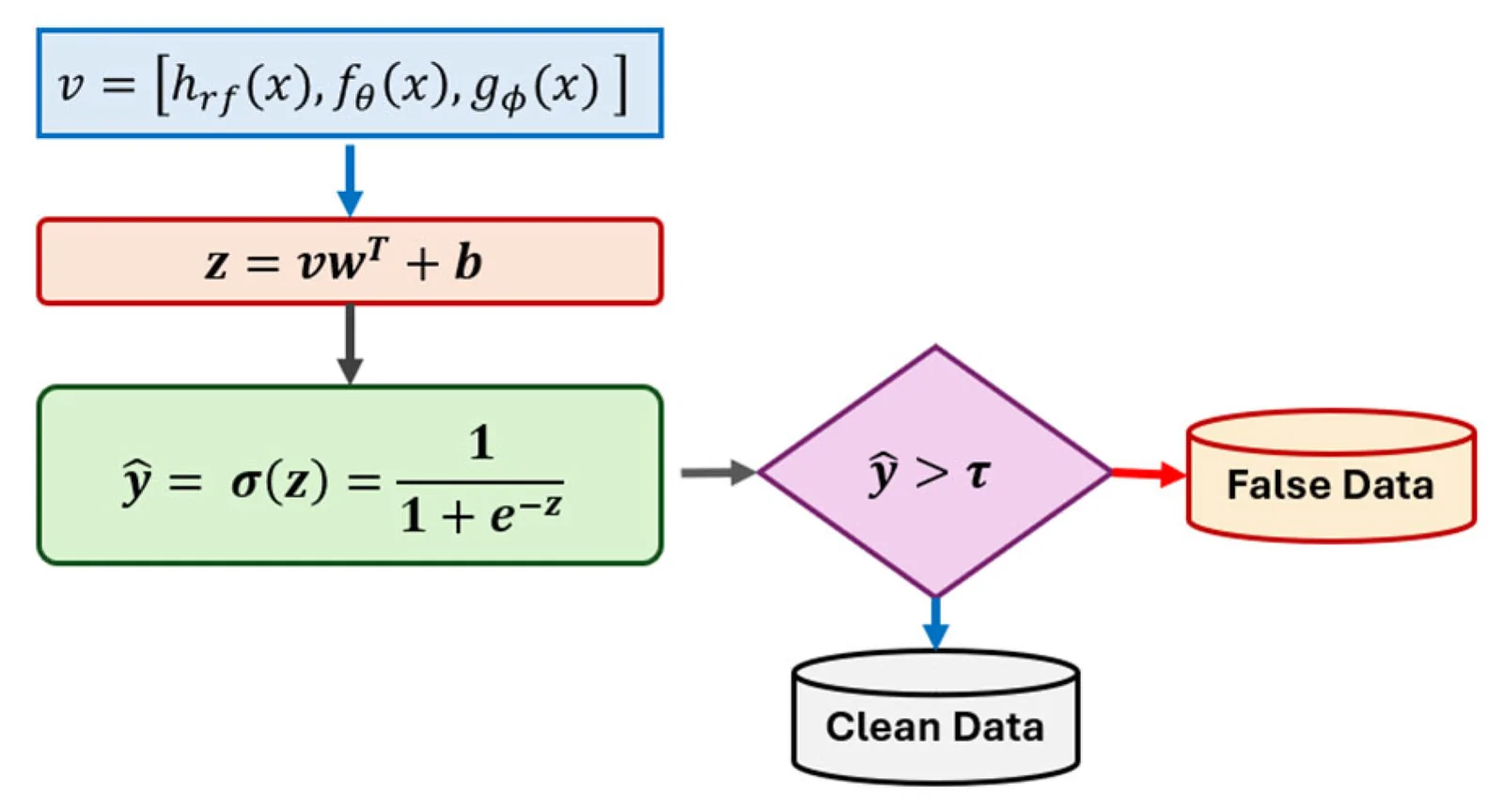
Logistic Regression meta-classifier for decision-making.

Rather than relying on either RF or AE models independently, the probabilistic output of the RF classifier is combined with the latent and reconstructed representations learned by the AE. These complementary features are subsequently fused by the Logistic Regression (LR) meta-classifier, which learns an optimal decision boundary in the combined feature space. LR is a linear probabilistic classification model. In this study, the LR classifier serves as the final decision-making model by integrating complementary information extracted from the preceding stages. LR was selected as the meta-classifier due to its simplicity, low computational cost, and suitability for combining the learned representations from the AE and RF outputs. Since the meta-classifier operates on a low-dimensional feature space consisting of the RF prediction probability and AE latent features, LR provides an effective and lightweight fusion mechanism that is less prone to overfitting than more complex models such as XGBoost or DNNs [47]. The input to the LR is a combined feature vector v, which consists of the probabilistic output generated by the RF classifier, the latent representation learned by the AE encoder, and the reconstructed features produced by the AE decoder. The combined feature vector is defined as

(20) $$v=\left[h_{rf}\;(x),f_{\theta }\;\left(x\right),g_{\phi }\;(z)\;\right]\;$$

where RF $h_{rf}\;(x)\;$ denotes the RF probability output, $f_{\theta }\;\left(x\right)$ represents the latent feature vector extracted by the encoder, and $g_{\phi }\;(z)\;$ denotes the reconstructed feature vector generated by the decoder. Combining the discriminative information provided by the RF classifier with the compressed and reconstructed representations learned by the AE enables the LR classifier to leverage complementary information for more accurate classification. The LR classifier estimates the probability of an input sample belonging to the positive class based on the combined input feature v.

(21) $$p(y=1|v)=\sigma (w^{T}v+b)\;$$

where p(y=1∣v) denotes the probability that the input feature vector v belongs to the positive class, w is the weight vector, b is the bias term, and σ(.) is the sigmoid function. LR learns parameters by minimising the cross-entropy loss function, which measures the discrepancy between the predicted probabilities and the true class labels.

(22) $$minL\left(w,b\right)=\min\left\{-\frac{1}{N}\sum_{i=0}^{N}\left[y_{i}log(p_{i})+\left(1-y_{i}\right)log(1-p_{i})\right]\right\}\;$$

where $y_{i}$ is the true class label of the $i^{\mathrm{th}}$ training sample, $p_{i}$ is the probability predicted by the LR classifier, N denotes the total number of training samples, and w and b are the model parameters to be optimised. Minimising the binary cross-entropy loss enables the LR classifier to learn the optimal decision boundary for distinguishing between normal and attack samples. Figure 3 illustrates the decision-making process of the proposed stacking meta-classifier. The outputs of the base learners are fused into a meta-feature vector, which is processed by Logistic Regression to generate an FDIA probability score. The final classification is obtained by comparing the predicted probability with a predefined decision threshold.

## 5. Evaluation Procedures

This section describes the TON_IoT Dataset, the FDIA simulation process, and the data preprocessing steps.

### 5.1. TON_IoT Dataset

In this study, the TON_IoT dataset [48] is used to validate the proposed model. The name is derived from telemetry datasets of IoT and IIoT sensors, Operating systems datasets, and network traffic datasets to reflect the heterogeneous nature of the data sources. TON_IoT is considered by many researchers [24,49,50] as a new generation of IoT and Industrial IoT (IIoT) cybersecurity datasets designed to evaluate the performance of Artificial Intelligence (AI)-based security solutions. TON_IoT is a large-scale, multi-layer IoT and IIoT cybersecurity dataset developed from a realistic Industry 4.0 testbed, containing telemetry, network, and system logs with labelled cyberattacks, designed for evaluating AI-based intrusion detection and cybersecurity models [24]. TON_IoT was developed to capture the diversity, scale, and heterogeneity of contemporary IoT environments that existing datasets fail to capture [49,50]. It also includes a variety of modern attack scenarios [49].

The telemetry data was collected from seven IIoT devices: fridge, GPS tracker, motion light, garage door, Modbus, thermostat, and weather. The dataset was collected using a realistic IIoT testbed [28,48] that integrates both physical and simulated IoT/IIoT devices, including real smartphones, smart TVs, and ESP8266 weather sensors alongside simulated-based services such as fridge, GPS, and thermostat simulators, to generate realistic telemetry data for cybersecurity evaluation [50].

TON_IoT covers nine attack categories, including reconnaissance/scanning, MITM, Distributed/Denial of Service (DoS/DDoS), password cracking, backdoors, ransomware, and injection attacks. Although these attacks primarily represent network-layer intrusions and do not directly capture application-layer data manipulation that characterises FDIAs, some categories, particularly MITM and injection attacks, represent network-level mechanisms that can facilitate unauthorised data modification in IIoT environments. Therefore, in this study, TON_IoT is used as an IIoT security benchmark, while additional adversarial FDIA scenarios are generated to simulate false data manipulation behaviours, including random noise, gradual drift, replay, stealthy, correlated, and adversarial attacks.

### 5.2. FDIA Simulation

**Figure 4:**
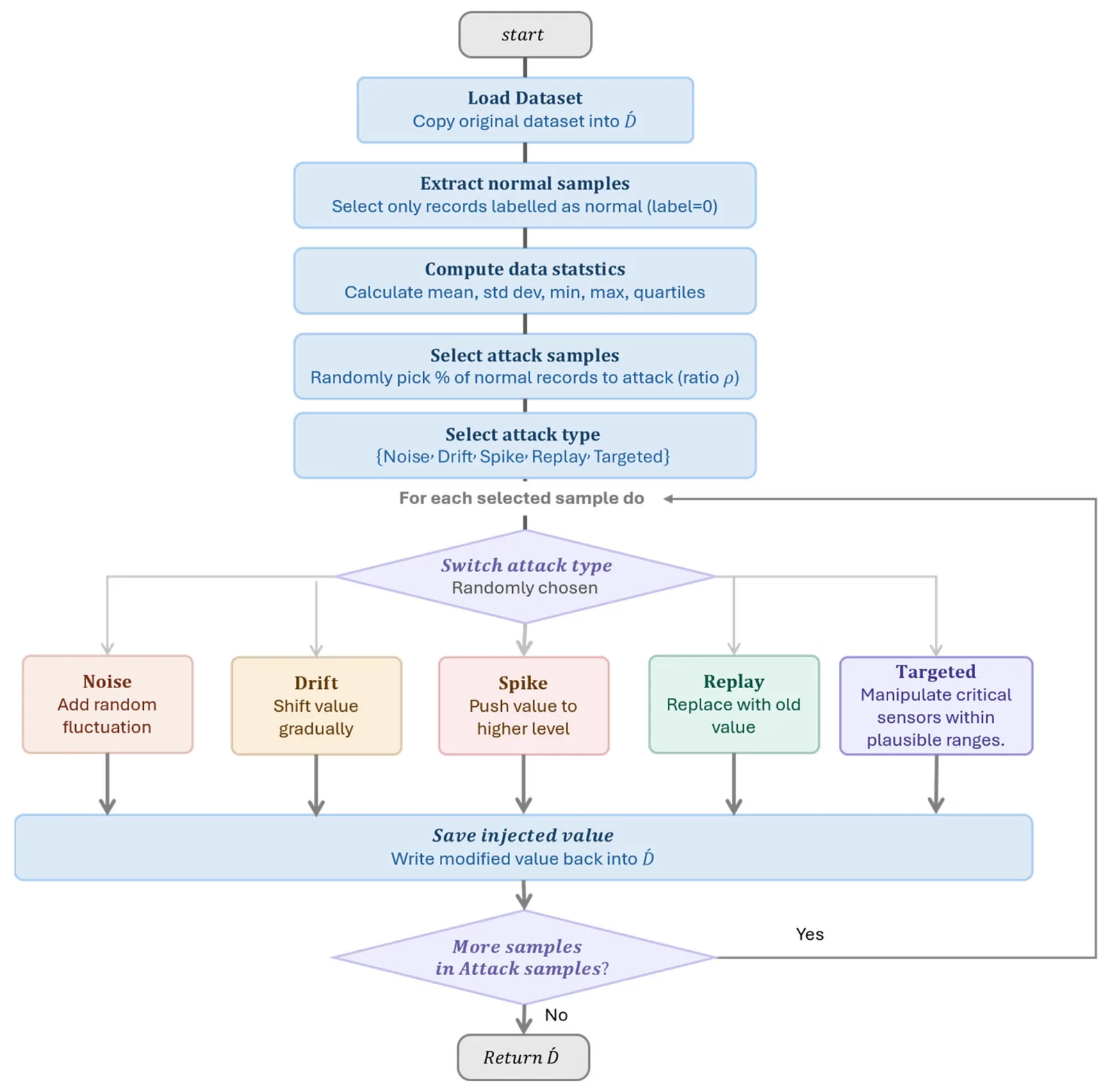
Flowchart of the implemented FDIA scenarios.

This section presents the implementation of the proposed FDIA Model described in Section 3. Figure 4 shows a simplified flowchart of the implemented FDIA Model. Algorithm 1 shows the detailed pseudocode of the implemented attack scenarios. As can be seen in Figure 4 and Algorithm 1, the FDIA model generates an adversarial dataset D′ by selecting normal records from a clean dataset D, analysing their statistical properties, and applying randomly chosen attack strategies (Noise, Drift, Spike, Replay, or Targeted) to modify selected samples. The altered records are then combined with the original data to produce the final adversarial dataset D′.

As illustrated in Algorithm 1 and Figure 4, five types of FDIAs were simulated and injected into randomly selected subsets of normal observations collected from the ICS environment represented in the TON_IoT dataset. The proposed attack generation framework is designed to produce adversarial samples that preserve the statistical characteristics of the original sensor measurements, thereby increasing the realism and stealthiness of the injected attacks. To achieve this, descriptive statistics, including the mean (μ), standard deviation (σ), minimum, maximum, and quartiles ($Q_{1}$ and $Q_{3}$), are computed for each sensor feature (Line 3). These statistics serve as constraints for generating perturbations that remain within the natural operating range of the monitored process instead of introducing arbitrary modifications. Next, a random subset of normal observations is selected according to the predefined attack ratio (Line 4). For each selected observation, one attack type is randomly chosen from the predefined attack set $\left\{\mathrm{Noise},\mathrm{Drift},\mathrm{Spike},\mathrm{Replay},\mathrm{Targeted}\right\}$ (Line 5).

The Noise attack is implemented by adding Gaussian perturbations to selected sensor features (Lines 10–14). Unlike conventional additive Gaussian noise, the perturbation is sampled from distributions centred around the interquartile ranges ($Q_{3}-\mu$ or $\mu -Q_{1}$) rather than zero. This implementation produces subtle deviations that remain consistent with the natural variability of the sensor measurements while avoiding obvious outliers, thereby increasing the attack’s stealthiness. The perturbation magnitude is constrained to 1.5IQR in the stealthy configuration. This constraint maintains the perturbations within a statistically plausible range of normal sensor variability, thereby reducing their likelihood of detection by conventional threshold- and distribution-based detectors. The attack is considered adversarial because the attacker is assumed to have knowledge of both the normal sensor-data distribution and the BDD thresholds and uses this knowledge to calibrate the perturbation magnitude to reduce the likelihood of detection while maintaining a meaningful deviation.
**Algorithm 1:** FDIA Attack Model**Input:** dataset D, random seed s, and attack ratio ρ **Output:**
 $\mathrm{adversarial}\;\mathrm{dataset}\;D^{\prime }$
1:  $\mathrm{Copy}\;\mathrm{sorted}\;\mathrm{dataset}\;D^{\prime }$$\overset{copy}{\leftarrow }\;D$ 2:  $\mathrm{Extract}\;D_{n}\overset{x\;\in \;D}{\leftarrow }\;x\;:label(x=0)$ 3:  Compute μ,σ,min, max, quartiles s ←st(D) 4: $Attack\_samples\leftarrow Random\_Samples(D_{n}\;,\left|D_{n}\right|\;\times \;\rho )$ 5:  $Stealthy\_Adversarial\_Attack\_Types\;(A)\leftarrow \;\left\{noise,\;drift,\;spike,\;replay,\;targeted\right\}\;$ 6:  For each i ∈ Attack_Samples do: 7:  Attack←Random(A) 8:  $Switch\;\left(Attack\right)$: 9:   Case noises: 10:    for each f ∈ features_set_1 11:     $z_{a}=f_{\left(x\right)}+\;\delta \;:\;\delta \in N\left(IQR,\;\sigma_{a}^{2}\right)$ 12:     $D^{\prime }$$\overset{update\;f(x)}{\leftarrow }\;$$z_{a}$ 13:    end for 14:   Case drift: 15:    for each f ∈ features_set_2 16:     $D^{\prime }$$\overset{update\;f(x)}{\leftarrow }z_{a}=f_{\left(x\right)}+\delta \;:\;\delta =\sigma \times \alpha$ 17:    end for 18:   case spike: 19:    for each f ∈ features_set_3 20:     $D^{\prime }$$\overset{update\;f(x)}{\leftarrow }z_{a}=f_{\left(x\right)}+\;\delta \;:\;\delta =\;IQR+\sigma \times \alpha$ 21:    end for 22:   case replay: 23:    for each f ∈ features_set_4 24:     $D^{\prime }$$\overset{update\;f(x)}{\leftarrow }z_{a}=random(f_{\left(x\right)})$$\;:\;\left|f_{\left(x\right)t}-\;z_{a}\right|\leq \delta$ 25:    end for 26:   case targeted: 27:    for each f ∈ features_set_5 28:     R(f)=max(f)−min(f) 29:     $D^{\prime }$$\overset{update\;f(x)}{\leftarrow }z_{a}=f_{\left(x\right)}+\;\alpha \times R(f)\;$ 30:    end for 31:  end case 32: end for

The Drift attack is implemented by adding Gaussian-distributed offsets to the original sensor values (Lines 15–18). The perturbation gradually shifts the sensor measurements toward a new operating point without introducing abrupt deviation. The drift magnitude is sampled from a uniform distribution between (0.5σ) and (2σ), where σ denotes the standard deviation of the corresponding sensor feature. This constrains the perturbation according to the sensor’s statistical variability, thereby maintaining stealthiness. The Spike attack is implemented by introducing an instantaneous perturbation proportional to the feature’s standard deviation (Lines 19–22). The perturbation is defined as (IQR+σ×α), where α ∈U(0.5,2) combining the feature’s interquartile range with a standard-deviation component scaled by a uniformly distributed factor between 0.5 and 2. This statistical calibration adapts the perturbation magnitude to the characteristics of each sensor, making the attack stealthy by maintaining consistency with the normal measurement distribution while generating a sudden transient deviation. The resulting spike is further constrained by the normal-value range, allowing a meaningful instantaneous deviation while reducing the likelihood of detection by simple range-based checks.

The Replay attack is implemented by replacing the current sensor reading with a randomly selected historical value from the same feature (Lines 23–26). The replayed value is selected such that the absolute difference between the current sensor value $f_{\left(x\right)t}$ at time step (t) and the selected historical value $z_{a}$, $\left|f_{\left(x\right)t}-\;z_{a}\right|$, is constrained by δ=1.5IQR. This constraint δ ensures that the replayed measurement remains close to the current sensor value and within a statistically plausible range, thereby contributing to the stealthiness of the attack. Since the substituted values originate from legitimate observations, the resulting measurements preserve the underlying statistical distribution of the sensor data while emulating replay attacks in which previously recorded measurements are retransmitted to conceal the current system state. Consequently, replay attacks are particularly difficult to distinguish from normal operating conditions. The Targeted attack is implemented by modifying selected critical features according to each feature’s dynamic range (Lines 27–31). In this study, the attack targets Modbus function-code registers, as these features represent control-related measurements that can influence the monitored process. For each selected feature, the perturbation is scaled according to its observed normal operating range, calculated as the difference between the maximum and minimum values of the feature. The feature value is then increased by a proportion of this range, where the scaling factor is randomly selected from a uniform distribution between 0.3 and 0.8. Scaling the perturbation by the feature range ensures that the injected deviation is proportional to the physical operating limits of each sensor, allowing the attacker to manipulate critical measurements while maintaining physically plausible values. This strategy emulates deliberate attacks aimed at influencing the control process without generating statistically implausible sensor readings.

The attacks implemented in Algorithm 1 are designed as stealthy and adversarial FDIA scenarios, with the injected perturbations constrained to remain within a statistically plausible range of each sensor’s normal operating behaviour. Specifically, the attack configurations use feature-specific properties, including the IQR, standard deviation, and observed normal operating range, to constrain the magnitude of the injected values. These constraints are applied across the different attack morphologies to ensure that the manipulated measurements remain within statistically plausible ranges while still introducing meaningful deviations from the original observations. Consequently, the attacks are less likely to be detected by conventional threshold- and residual-based BDD mechanisms that rely primarily on statistical deviations from normal measurements.

The attacks are considered adversarial because the threat model assumes that the attacker has knowledge of the detection boundaries employed by the BDD mechanism and deliberately calibrates the injected perturbations to reduce the likelihood of detection. This reflects a detector-aware adversary that exploits knowledge of the deployed detection mechanism when crafting the attacks. However, the proposed attacks do not employ machine-learning-based optimisation techniques, such as gradient-based methods or generative model-based approaches. Such optimisation-based adversarial attacks are beyond the scope of this study and are considered a direction for future work.

For comparison, a corresponding set of basic FDIA was implemented using the same five attack morphologies but without the statistical constraints and detector-aware calibration adopted in the proposed threat model. Table 1 summarises the attack configurations and feature-selection rationale for both the basic and proposed stealthy–adversarial FDIA scenarios. The proposed attacks use sensor-specific statistical characteristics and assume knowledge of the BDD boundaries to constrain the injected values and reduce their detectability. This provides a controlled comparison between statistically naive and detector-aware FDIA scenarios while maintaining consistent attack morphologies.

As shown in Table 1, the features targeted by each attack morphology in the proposed threat model were selected according to their physical characteristics and operational roles within the TON_IoT environment. This domain-driven selection ensures that each attack is applied to measurements for which the corresponding manipulation is physically meaningful and operationally realistic. In contrast, the basic FDIA uses randomly selected features without applying these domain-specific selection criteria. Thus, the two scenarios differ not only in the degree of statistical constraint applied to the perturbations but also in the feature-selection strategy, with random feature selection used for the basic attacks and operationally informed selection used for the proposed stealthy–adversarial attacks.

### 5.3. Dataset Preprocessing

In the dataset preprocessing stage, all instances collected from IoT devices were first sorted chronologically according to their timestamps to preserve temporal consistency. To prevent data leakage, the original TON_IoT dataset was first divided into independent subsets, with 50% allocated for training the base classifiers and the remaining 50% retained as hold-out data. All subsequent processing and FDIA generation were performed separately within each partition using only the data available in the corresponding subset.

The ordered data was then replayed in a controlled manner to emulate real-time data acquisition at the edge layer. To construct time-epoch representations, the data was segmented into fixed-length intervals. Within each epoch, feature aggregation was performed using min–max statistics, conditioned on the associated labels. In attack-dominant epochs, malicious samples were allowed to dominate the aggregated representation to reflect realistic intrusion scenarios where adversarial behaviour governs system dynamics during active compromise periods.

FDIA instances were introduced into segments following the occurrence of an original network attack in the dataset to simulate realistic adversarial manipulation of system observations. This injection strategy was designed to capture different attack stages, including reconnaissance, scanning, and credential compromise, such as password cracking, as well as injection attacks such as cross-site scripting and SQL injection attacks. Additionally, a subset of FDIA samples was independently injected without direct correlation to prior attack stages to emulate stealthy and adversarial behaviours. This approach ensures diversity in attack patterns, including both staged and opportunistic injection scenarios to improve the model’s ability to generalise across different attack scenarios.

To generate the FDIA samples, 10% of the normal samples from each partition were selected as the FDIA generation pool, corresponding to the attack ratio ρ in Algorithm 1. As the number of normal samples substantially exceeded the number of originally labelled attack samples, using the full generation pool could produce an excessive number of synthetic FDIA instances. Therefore, the proportion of FDIA samples in both the training and test sets was capped at 20% of the total attack population. Consequently, approximately 80% of the attack samples correspond to the original attacks in the dataset, while up to 20% consist of generated FDIA samples. This approach preserves the original attack distribution while maintaining a controlled proportion of synthetic FDIA samples for evaluating detection performance.

The base classifiers were trained exclusively using the initial 50% training partition. The 50% hold-out partition was subsequently divided into two subsets: 60% (equivalent to 30% of the full dataset) was used to generate RF output probabilities and AE reconstruction errors for training the Logistic Regression meta-classifier, while the remaining 40% (equivalent to 20% of the full dataset) was reserved for the final evaluation of the complete detection framework. Thus, the base classifiers, LR meta-classifier, and final evaluation were performed using separate data subsets, thereby preventing information leakage across the evaluation stages.

## 6. Results and Discussion

**Figure 5:**
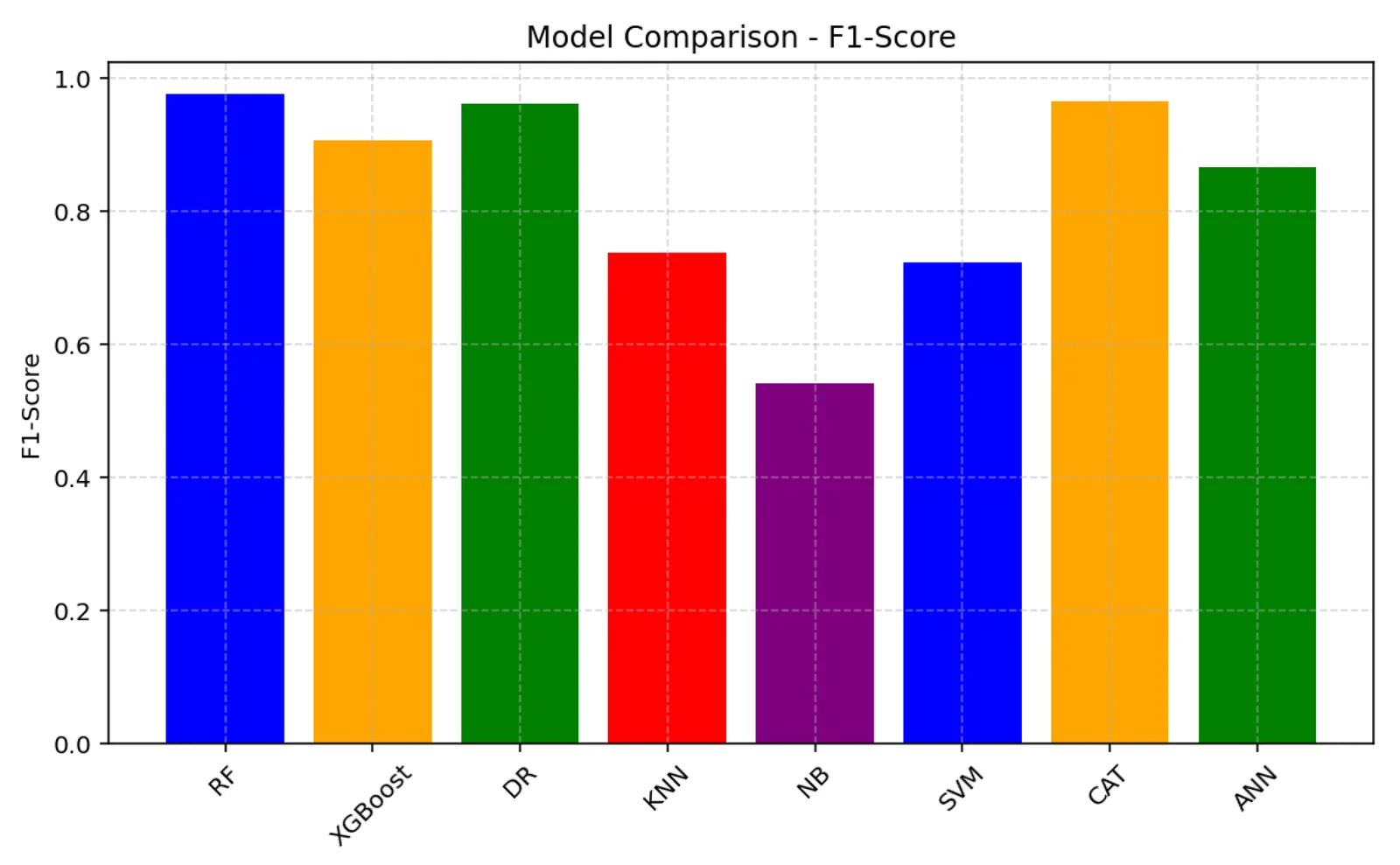
Performance comparison of the evaluated machine learning models in terms of the F1-score under Basic FDIAs.

**Figure 6:**
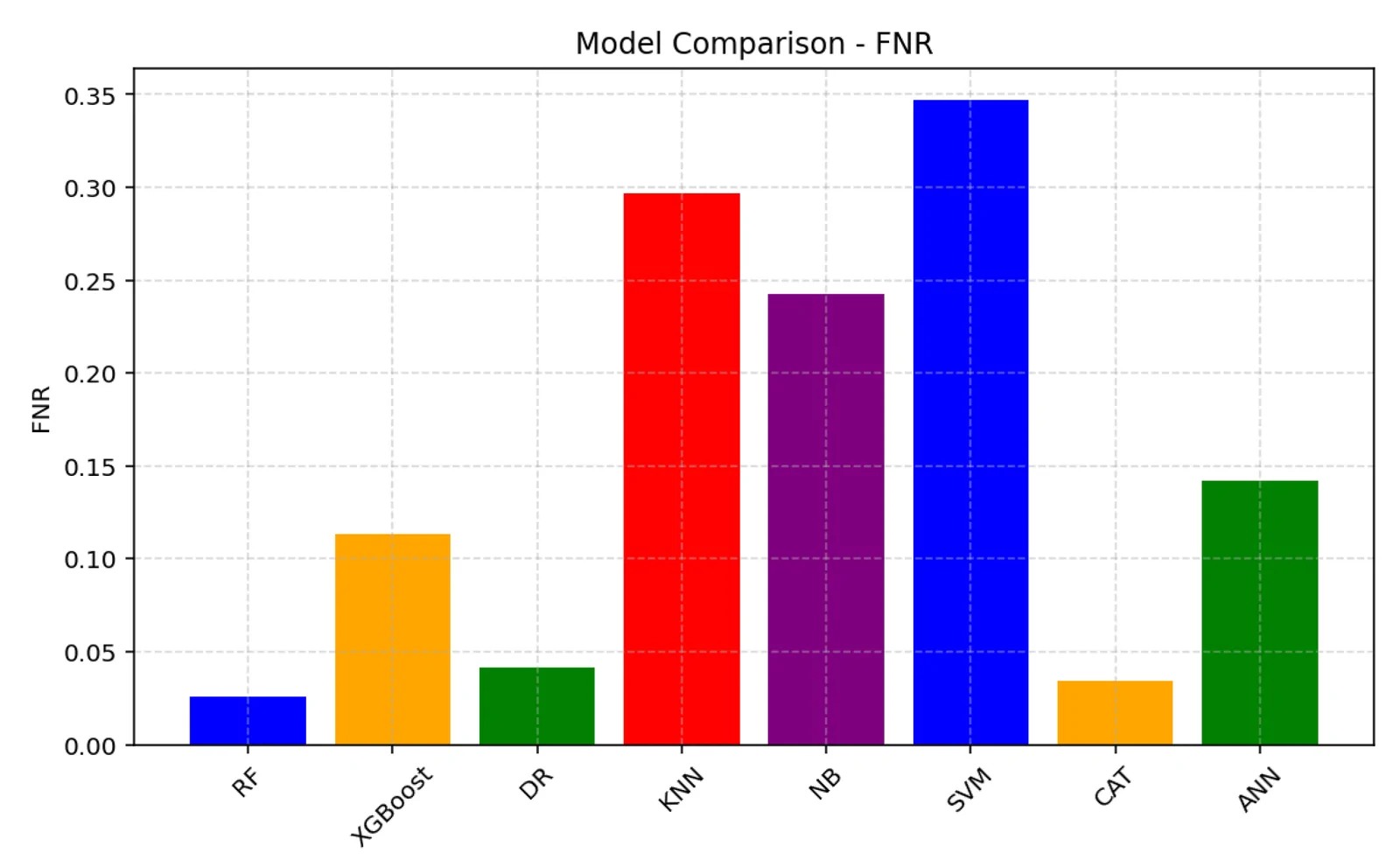
Performance comparison of the evaluated machine learning models in terms of the False Negative Rate (FNR) under Basic FDIAs.

Table 2 presents the performance evaluation of several machine learning classifiers trained on the TON_IoT dataset, where 20% of the attack samples were generated as basic false data injection attacks, while the remaining 80% consisted of original TON_IoT intrusion samples. The results demonstrate that ensemble-based methods significantly outperform other classifiers. This improvement can be attributed to their ability to capture complex nonlinear relationships and diverse attack patterns within high-dimensional industrial sensor data. Among all evaluated models, the RF classifier achieved the best overall performance in terms of F-measure with an F1-score of 97.47%, as illustrated in Figure 5. RF also achieved the lowest false positive rate, 0.42%, and False Negative Rate (NFR) of 2.6%, as shown in Figure 6. These results demonstrate the capability of RF to effectively distinguish between normal and malicious observations while minimising both false alarms and missed attacks. This finding is consistent with previous studies [10,11,22], where RF has been frequently reported as a high-performing classifier for intrusion detection and cyber-physical system security applications.

The other ensemble-based models, including CAT and DR, also achieved competitive performance, with F1-scores of 96.41% and 96.07%, respectively, as shown in Figure 5. CAT achieved a higher recall (96.58%) compared with RF, which indicates strong attack detection capability; however, RF maintained a better balance between false positive and false negative errors. In contrast, traditional classifiers such as KNN, SVM, and NB demonstrated lower detection performance, particularly in terms of recall and FNR. For example, SVM and KNN obtained FNR values of 34.68% and 29.65%, respectively, which indicates that a significant proportion of FDIA samples were incorrectly classified as normal, as shown in Figure 6. NB achieved the lowest overall performance, with an F1-score of 54.04% and an FPR of 17.97%, which can be attributed to its assumption of feature independence that does not adequately represent the correlations among industrial sensor measurements.

**Figure 7:**
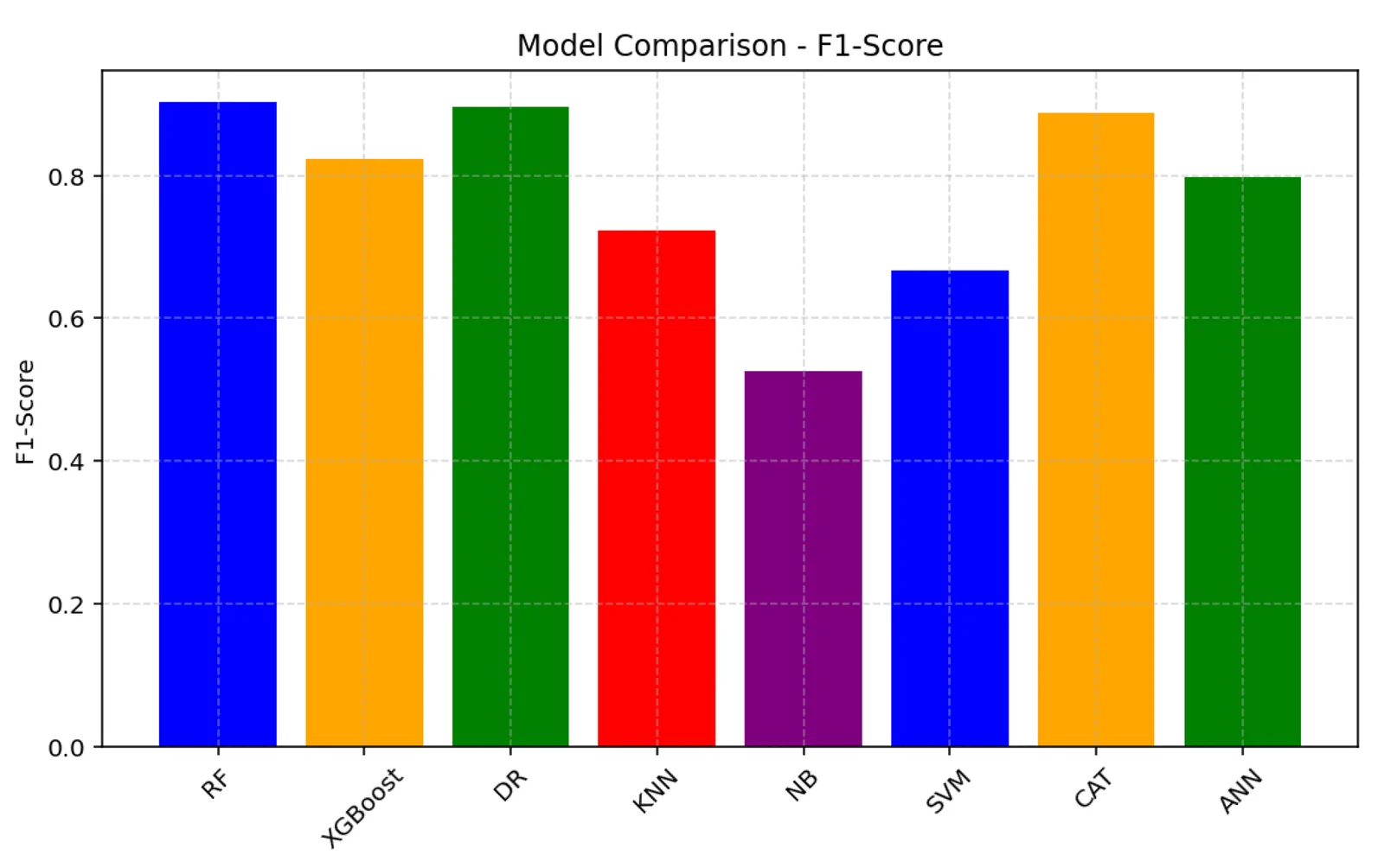
Performance comparison of the evaluated machine learning models in terms of the F1-score under Stealthy FDIAs.

**Figure 8:**
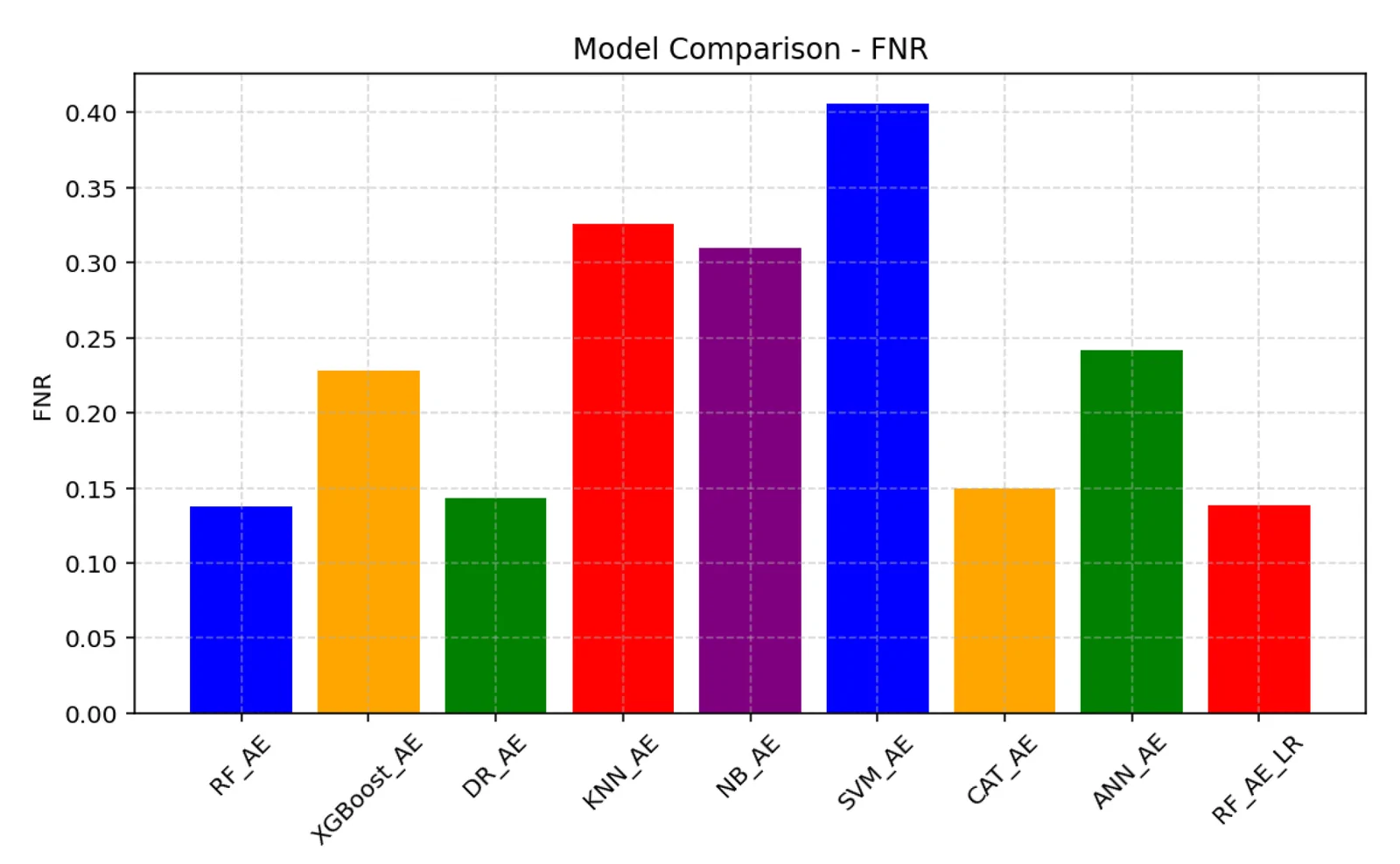
Performance comparison of the evaluated machine learning models in terms of the False Negative Rate (FNR) under Stealthy FDIAs.

Table 3 presents the performance evaluation of the same machine learning models under a more challenging intrusion scenario, where 20% of the total attack samples are generated as stealthy and adversarial false data injection attacks and 80% consist of original TON_IoT intrusion samples. This setting is designed to evaluate the robustness of intrusion detection models against more subtle and intentionally crafted attacks that aim to evade detection mechanisms. Figure 7 and Figure 8 further illustrate the comparison of the evaluated models in terms of F1-score and False Negative Rate (FNR), respectively. Compared with the basic FDIA scenario presented in Table 2, the results in Table 3 show a noticeable performance degradation compared to simpler attack scenarios with all tested classifiers, particularly in terms of recall and false negative rate, highlighting the increased difficulty of detecting stealthy FDIA samples. This reduction is expected because stealthy FDIAs are designed to generate subtle perturbations that preserve the statistical characteristics of legitimate sensor measurements, thereby reducing the separability between normal and malicious observations. Despite this, ensemble-based methods still demonstrate superior performance compared to conventional machine learning techniques. As shown in Figure 7, the RF classifier achieved the highest F1-score (90.24%) among all evaluated models, followed by DR (89.67%) and CAT (88.71%). However, its recall of 83.08% indicates that a portion of stealthy attacks still go undetected, reflected in the false negative rate of 16.92%, as shown in Figure 8. This suggests that while RF remains robust, stealthy FDIA significantly affects its detection sensitivity.

Table 4 presents the performance of the proposed hybrid models, where the AE is integrated with each machine learning classifier to improve feature representation under the stealthy FDIA scenario. As presented in Table 4, the incorporation of the AE improved the detection performance of most classifiers, indicating that the learned latent feature representations provide better discrimination between normal observations and stealthy attack samples. Among the evaluated models, RF_AE achieved the best overall performance, obtaining the highest F1-score (91.28%) and the lowest FNR (13.74%), while maintaining a low FPR of 0.58%. DR_AE and CAT_AE also demonstrated competitive performance, achieving F1-scores of 90.62% and 89.78%, respectively.

Table 5 presents a comparative performance evaluation of three related models: RF, autoencoder-enhanced RF (RF_AE), and RF_AE_LR under stealthy and adversarial FDIA. The objective of this comparison is to assess the impact of feature representation learning via autoencoders and hybrid classification strategies on improving detection robustness against sophisticated FDIAs patterns. The results show a consistent improvement in performance as model complexity increases. The progressive enhancement from RF to RF_AE and finally RF_AE_LR proves consistent performance gains, particularly in detection capability. The most significant improvement is observed in recall, which increases by 5.17 percentage points. This improvement reflects a substantially enhanced ability to detect FDIA instances. The overall performance in terms of F1-score improves by 2.39 percentage points, reflecting a better balance between precision and recall. LR layer effectively refines the final classification by exploiting the discriminative latent representations learned by the AE and the decision patterns produced by the RF classifier.

**Figure 9:**
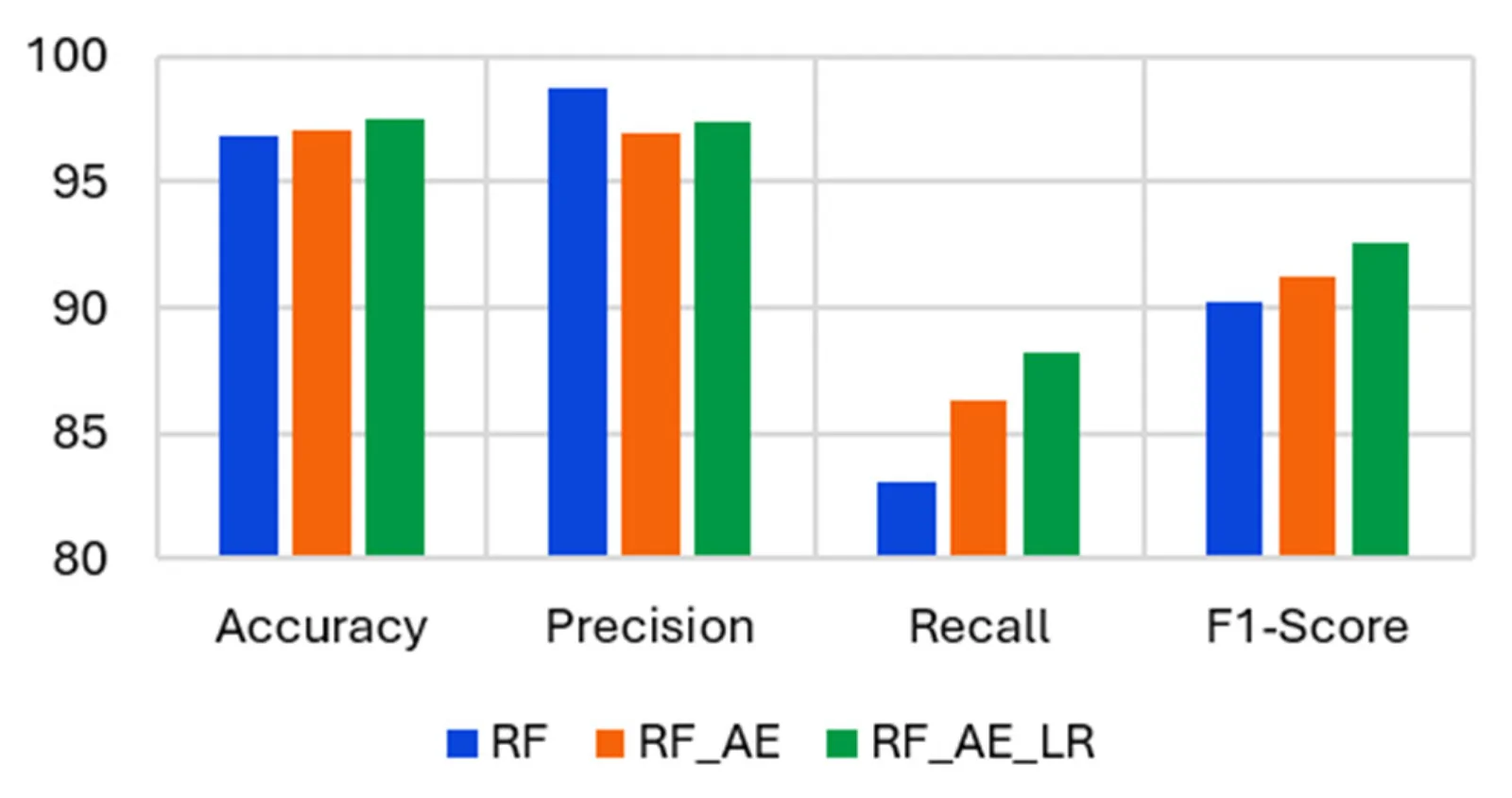
Performance comparison of the proposed Hybrid Models in terms of the F1-score under Stealthy FDIAs.

**Figure 10:**
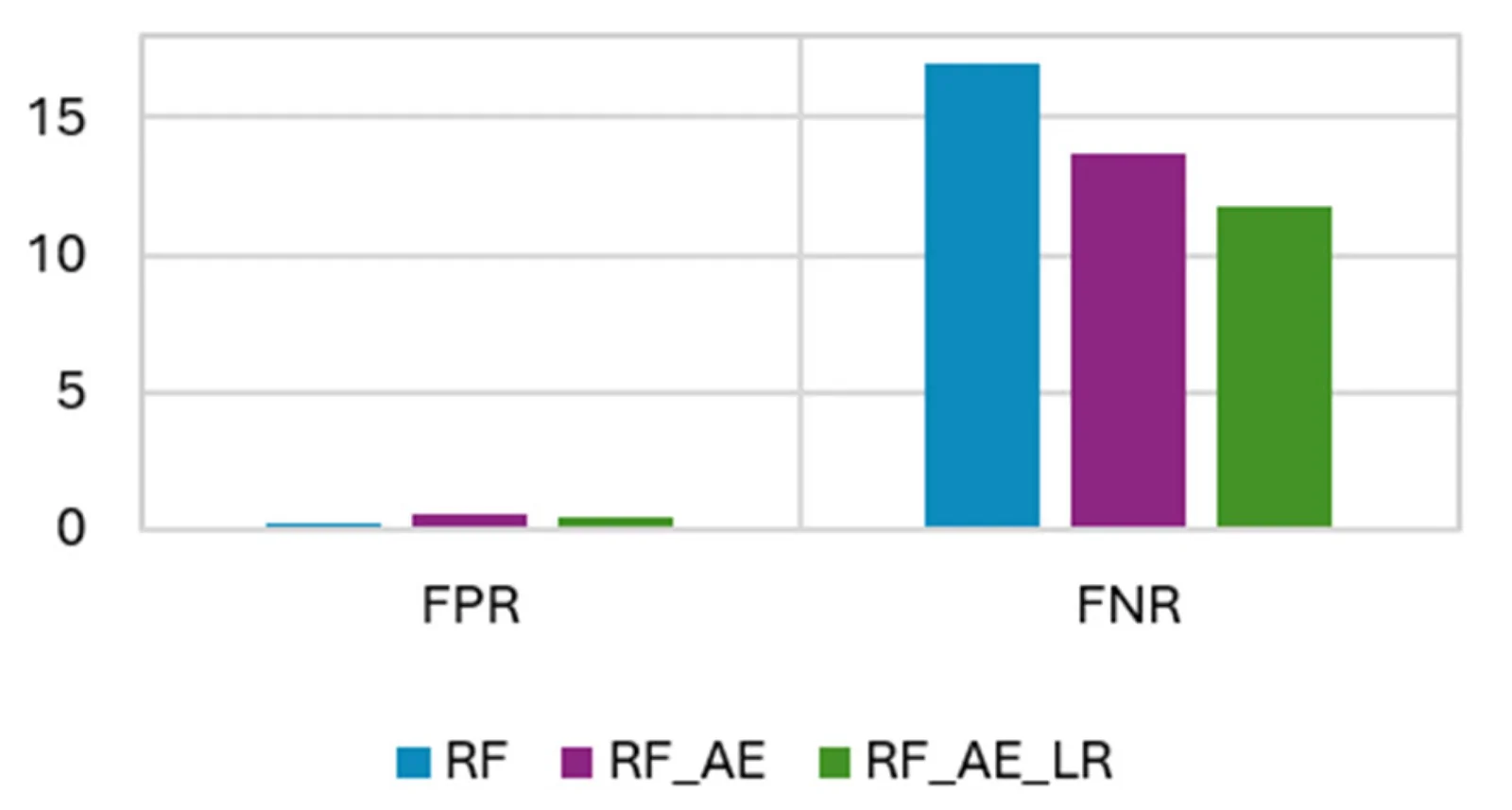
Performance comparison of the proposed Hybrid Models in terms of the False Negative Rate (FNR) under Stealth FDIAs.

As shown in Table 5, Figure 9 and Figure 10, while the standalone RF classifier achieved high precision (98.75%), its recall was limited to 83.08%, indicating that some stealthy FDIA samples remained undetected. This behaviour is expected because stealthy attacks are intentionally generated to preserve the statistical characteristics of legitimate sensor measurements, reducing the separability between normal and malicious observations in the original feature space. Incorporating the autoencoder (RF_AE) improved recall to 86.26% and reduced the FNR from 16.92% to 13.74%, suggesting that the learned latent representations enhance the separability between normal and attack samples. By encoding the correlations among multiple sensor measurements into a compact representation, the autoencoder increases the discriminative information available to the RF classifier, enabling it to identify attack samples whose statistical deviations are too subtle to be reliably detected using the original features. The proposed RF_AE_LR model further increased recall to 88.25% and achieved the highest F1-score (92.63%) while reducing the FNR to 11.75%. This improvement indicates that the LR layer effectively refines the final classification of ambiguous samples in the transformed feature space.

As shown in Table 2, Table 3, Table 4 and Table 5, FDIA significantly challenges intrusion detection systems by reducing recall and increasing false negatives. The attacks presented in the TON_IoT are learnable, as demonstrated by the overall performance of most classifiers shown in Table 2. However, a significant degradation in performance occurs when stealthy and adversarial attacks are injected into the test dataset, as shown in Table 3. This degradation highlights the difficulty of detecting sophisticated FDIA variants and indicates that conventional classifiers may struggle to maintain reliable detection capability under realistic attack scenarios.

From an operational perspective, this reduction in detection capability is critical, as missed FDIAs correspond to undetected data integrity violations that may propagate through the system without immediate detection. To address this limitation, the proposed hybrid architecture combines RF with an AE to enhance feature representation and improve attack discrimination. The AE extracts latent representations from the input data, enabling the RF classifier to identify complex attack patterns that may not be sufficiently distinguishable using the original feature space alone. Consequently, the RF-AE model improves recall by 3.18 percentage points and reduces the false negative rate from 16.92% to 13.74%.

Although the integration of AE improves the detection capability of the RF classifier, it introduces a slight reduction in precision, decreasing from 98.75% to 96.92%. To address this trade-off, LR is incorporated as a metaclassifier to combine the outputs of the RF and AE components. The resulting RF-AE-LR model compensates for the precision reduction and achieves a precision of 97.46%, while maintaining the improved detection capability provided by the hybrid architecture.

Although the proposed RF_AE_LR model achieves improved FDIA detection performance while maintaining a low false-positive rate of 0.49%, its detection rate of 88.25% remains insufficient for reliable deployment in IIoT environments. This limitation highlights the vulnerability of the current detection framework to false data injection attacks. Additional limitations are associated with the TON_IoT dataset itself. In the TON_IoT dataset, sensor measurements are not temporally coordinated. Although timestamps are provided, samples generated from different sensors are treated as independent observations rather than as time-ordered sequences representing the continuous operation of an IIoT system. Consequently, the temporal dependencies and dynamic characteristics of sensor measurements are not fully captured, which may limit the ability of detection models to learn realistic attack behaviours.

Many FDI attacks are characterised by their temporal evolution rather than network behaviour. Both RF and AE models process each sample independently, creating a fundamental vulnerability to stealthy FDIAs that gradually shift the system’s normal operating conditions. While the AE can signal abnormal behaviour through elevated reconstruction error, this mechanism is only effective when the cumulative drift exceeds the boundaries of normal reconstruction noise, leaving slow incremental attacks below that threshold undetected. He et al. [4] demonstrated that treating FDIA detection as a time-series problem rather than a point-wise classification problem can yield substantially higher detection rates. However, the work in [4] was trained using basic FDIAs simulated specifically for the IEEE 118-bus system, which limits its generalisability to broader attack scenarios and real-world IIoT environments. Future work should explore architectures that explicitly incorporate temporal and time-series dependencies, such as LSTM networks or TCNs, to address the inherent limitation of the RF component in detecting temporally defined FDIA variants, such as ramp and slow-drift attacks. Furthermore, datasets specifically designed for FDIA detection, such as UKMNCT_IIoT_FDIA [17] and SWaT [51], should be studied for evaluating the proposed approach beyond the representational constraints of TON_IoT.

Although the proposed hybrid model introduces additional computational steps compared with a standalone RF classifier, inference remains lightweight because the autoencoder performs a single forward pass and Logistic Regression has linear computational complexity. Nevertheless, evaluating end-to-end inference latency, memory consumption, and throughput on representative IIoT edge hardware is beyond the scope of this work and will be investigated in future research.

## 7. Conclusions and Future Work

This study investigated the challenges associated with detecting stealthy and adversarial FDIAs in IIoT environments. Unlike many existing FDIA detection studies that primarily focus on conventional attack scenarios or point-wise classification problems, this work extends existing research by evaluating intrusion detection models under more realistic attack conditions and proposing a hybrid RF-AE-LR framework that combines supervised classification and unsupervised feature learning. RF creates complex and non-smooth feature boundaries that make stealthy false data injection patterns more difficult to evade and easier to distinguish once distributional deviations emerge in the observed data. A deep autoencoder learns compact and high-level latent representations of normal system behaviour, enabling it to capture subtle deviations and hidden anomalous patterns introduced by stealthy FDIA that may not be easily distinguishable in the original feature space. The experimental results demonstrate that conventional classifiers can achieve high performance under standard TON_IoT attack distributions; however, their effectiveness decreases significantly when exposed to stealthy and adversarial FDIA variants. The proposed hybrid approach improves detection performance by 2.39 percentage points in terms of F1-score. This demonstrates the effectiveness of feature representation learning using autoencoders and hybrid classification strategies for detecting sophisticated FDIA patterns.

The main limitation of the proposed approach is that both the RF and AE components process individual samples independently and do not explicitly capture temporal dependencies between consecutive observations. This limitation may reduce the capability of the model to detect stealthy FDIA scenarios that evolve gradually over time and cause subtle deviations from normal system behaviour, such as ramp and slow-drift attacks. Future work should investigate architectures that explicitly model temporal and sequential dependencies, such as LSTM networks or TCN, to overcome the inherent limitation of the RF component in identifying temporally evolving FDIA variants, including ramp and slow-drift attacks. A further limitation is that the current FDIA generation framework relies on statistically bounded perturbations and assumed knowledge of the BDD decision boundaries. Optimisation-based adversarial attacks, including gradient-based methods and generative approaches, were not considered. Incorporating such attack-generation strategies would provide a more comprehensive evaluation of the framework against advanced adversarial threats. Although the proposed framework achieves higher recall and F1-score, this improvement involves a slight increase in the false positive rate compared with the RF baseline, highlighting the need to balance detection effectiveness and operational efficiency in practical IIoT deployments. Furthermore, future evaluations will include stratified robustness analyses across different FDIA intensities, attack ratios, and variants to assess the resilience of the proposed framework under diverse attack conditions.

## Figures and Tables

**Table 1:** Parameter configurations and target features for the implemented FDIAs.

**Attack**	Basic	Stealthy & Adversarial	Features Set and Rationale
Noise	$\sigma_{a}=3IQR$	$\sigma_{a}=1.5IQR\;$	Environmental measurements: Temperature, humidity, and pressure—subject to measurement noise and random variation
Drift	α=3	α ∈U(0.5,2)	Thermal measurements: Fridge temperature and current temperature—typically exhibit gradual variations over time
Spike	α=3	α ∈U(0.5,2)	Physical process features: pressure and temperature—may exhibit sudden transient changes
Replay	δ=0	δ=1.5×IQR	All features: Full vector—to preserve the consistency of a legitimate historical observation
Targeted	α=1	$\alpha \in U\left(0.3,0.8\right)$	Modbus function-code features: (FC1, FC2, FC3, FC4)—represent ICS communication and control-related measurements associated with actuator operations.

**Table 2:** Performance evaluation under 20% basic FDIA and 80% TON_IoT intrusions.

Model	Accuracy	Precision	Recall	F1-Score	FPR	FNR
RF	99.26	97.53	97.40	97.47	0.42	2.60
XGBoost	97.31	92.70	88.67	90.64	1.20	11.33
DR	98.85	96.31	95.83	96.07	0.63	4.17
KNN	92.64	77.41	70.35	73.71	3.53	29.65
NB	81.11	42.01	75.75	54.04	17.97	24.25
SVM	92.65	80.88	65.32	72.27	2.65	34.68
CAT	98.94	96.24	96.58	96.41	0.65	3.42
ANN	96.09	87.33	85.79	86.55	2.14	14.21

**Table 3:** Performance evaluation under 20% stealthy FDIA and 80% TON_IoT intrusions.

Model	Accuracy	Precision	Recall	F1-Score	FPR	FNR
RF	96.86	98.75	83.08	90.24	0.22	16.92
XGBoost	94.46	92.85	74.00	82.36	1.21	26.00
DR	96.67	97.95	82.67	89.67	0.37	17.33
KNN	91.40	82.71	64.20	72.29	2.84	35.80
NB	79.19	43.67	66.02	52.57	18.02	33.98
SVM	90.21	82.09	56.20	66.72	2.59	43.80
CAT	96.36	96.77	81.89	88.71	0.58	18.11
ANN	93.57	88.53	72.62	79.79	1.99	27.38

**Table 4:** Performance evaluation of the proposed hybrid model under 20% stealthy FDIA and 80% TON_IoT intrusions.

Model	Accuracy	Precision	Recall	F1-Score	FPR	FNR
RF_AE	97.12	96.92	86.26	91.28	0.58	13.74
XGBoost_AE	94.74	91.31	77.23	83.68	1.56	22.77
DR_AE	96.90	96.16	85.68	90.62	0.72	14.32
KNN_AE	91.69	81.83	67.39	73.91	3.17	32.61
NB_AE	79.46	44.35	69.04	54.01	18.34	30.96
SVM_AE	90.50	81.13	59.42	68.60	2.93	40.58
CAT_AE	96.62	95.06	85.06	89.78	0.93	14.94
ANN_AE	93.84	87.28	75.80	81.14	2.34	24.20

**Table 5:** Performance comparison of the proposed hybrid model.

Model	Accuracy	Precision	Recall	F1-Score	FPR	FNR
RF	96.86	98.75	83.08	90.24	0.22	16.92
RF_AE	97.12	96.92	86.26	91.28	0.58	13.74
RF_AE_LR (the proposed)	97.54	97.46	88.25	92.63	0.49	11.75

## Data Availability

The dataset used in this study is publicly available online at https://research.unsw.edu.au/projects/toniot-datasets (accessed on 9 August 2026).
